# Refusal in physical education—teachers’ strategies and utilization of digital media

**DOI:** 10.3389/fspor.2025.1576792

**Published:** 2025-04-10

**Authors:** Pierre Meinokat, Katja Reimers, Ingo Wagner

**Affiliations:** ^1^ Institute for School Pedagogy and Didactics (ISD), Karlsruhe Institute of Technology (KIT), Karlsruhe, Germany; ^2^ Institute of Fine Arts, Music and Sports, Ludwigsburg University of Education, Ludwigsburg, Germany

**Keywords:** physical education refusal, digitalization, classroom management, classroom disruptions, interview, teacher, digital media

## Abstract

**Introduction:**

Physical education refusal (PER) is a subject-specific form of classroom disruption within physical education (PE). PE teachers are tasked with managing PER effectively to improve learning outcomes and protect their well-being. Teacher interventions occur across institutional, classroom, and relationship dimensions. However, existing research has not yet adequately addressed the increasing role of digitalization.

**Methods:**

Therefore, this interview study explores the potential of digital media by investigating PE teachers' strategies for dealing with PER (RQ1) and their use of digital media in this process (RQ2).

**Results:**

Findings show, for the first time internationally, an empirical basis for connecting the dimensional framework to reported strategies, hereby confirming and enhancing existing research. Teachers tend to use strategies that are based in the classroom dimension where they seem to have the best possible influence. In terms of use of digital media, teachers prefer software solutions to deal with PER, supplemented, if possible and sensible, by hardware and methodical structuring.

**Discussion:**

Combining these findings into a preliminary model, this study lays the foundation for future research in dealing with PER in digitally based PE lessons.

## 1 Introduction

Minimizing classroom disruptions is essential for student academic success (1–3). However, classroom disruptions are frequent, occurring on average every 42 s (4). In physical education (PE), this issue is confirmed by Maddeh et al. (5), who report 1.2 disruptions per minute. These disruptions impede learning and, in addition, can negatively affect teacher well-being (6–8). Effective classroom management is crucial for addressing these disruptions. Dealing with students refusing to participate is a key challenge in PE classroom management (9). While this constitutes a known challenge, the ongoing digital transformation of education (10–12) requires investigating the untapped potential of digital media in addressing student PE refusal.

## 2 Theoretical framework

### 2.1 Classroom management and classroom disruptions

Kounin (13) introduced the concept of classroom management, initially focusing on student and group management. Currently, classroom management encompasses “the actions teachers take to create an environment that supports and facilitates both academic and social-emotional learning” [(14), 4]. Smooth classroom and lesson operation (15) is influenced by various factors (16). Although authors emphasize different aspects of classroom management, a common thread is the development of skills to create a productive and effective learning environment.

A crucial component of classroom management addresses classroom disruptions, defined as “behavior that seriously interferes with the teaching process, and/or seriously upsets the normal running of the classroom” [(17), 493]. Disruptions can manifest in various forms. While the integration of digital media has introduced new types of general classroom disruptions (42), the unique context of PE also presents distinct challenges (18).

### 2.2 Physical education refusal (PER)

School refusal is a broad term (19) encompassing resistance to school or specific classes. Physical education refusal (PER) describes this issue within PE. Wolters and Gebken [(9), 4] defined PER as “all behaviors in which the rejection of PE is expressed (from disrupting, sitting on the bench and forgetting sportswear to absenteeism)” (translated by authors). While this issue is not unique to the German educational system, international research on PER so far remains limited. Research using synonymous terms for PER such as *benchwarmers*, *reluctance*, *unwillingness* or simply *missing interest* (20) so far is not connected to the field of research on classroom management in PE.

**Figure 1:**
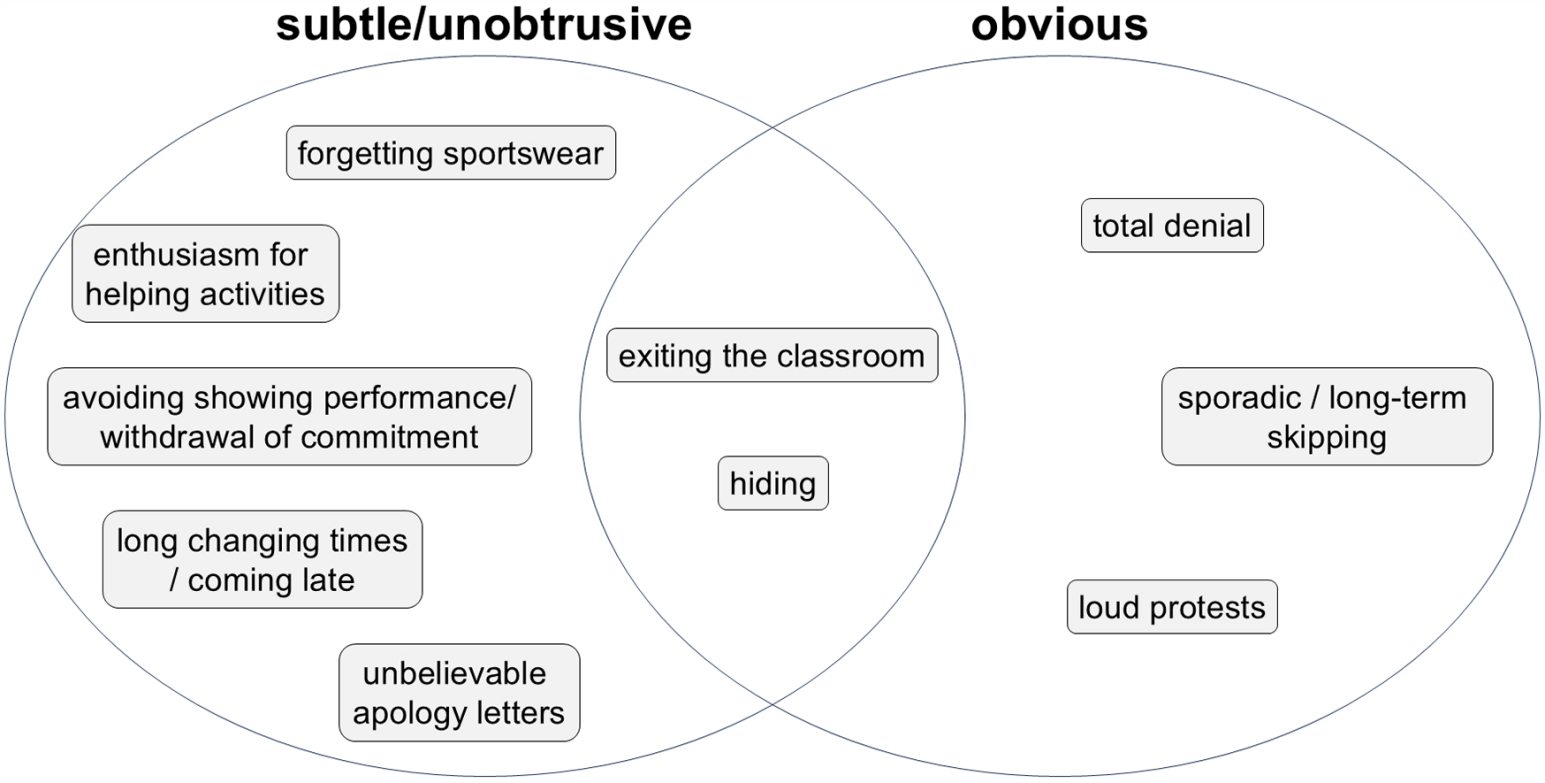
Examples for PER behaviors.

Kleiner and Reichel (21) categorized PER behaviors on a spectrum from subtle and unobtrusive to overt refusal. Behaviors described by other researchers (9, 22–25) fit within these categories. Subtle behaviors include forgetting sportswear, extended changing times, feigning enthusiasm for helping activities, avoiding performance displays, or providing implausible excuses. Overt behaviors include outright refusal, potentially with vocal protests, or repeated and extended absences. Some behaviors, like hiding or quietly leaving the classroom, can fall into either category. Figure 1 illustrates the state of research in this regard.

### 2.3 Teachers’ strategies regarding PER

In response to student PER behaviors, teachers employ strategies to manage these situations. Strategies are defined as the actions and practices teachers use to maintain or restore effective classroom management (26). Wolters and Gebken (9) categorized the reasons for PER as *in-school* or *out-of-school*. They argued that teachers and students have greater influence over in-school factors, leading to three dimensions for understanding PER reasons: *institutional*, *classroom*, and *relational* (between classroom stakeholders). Within this framework Frohn (20) is able to confirm these dimensions for primary school level through an interview study with students. While this creates a structural framework for understanding PER behavior other authors report strategies teachers use as a response to various situations in PE, including PER (9, 22–25, 27–31). Research on these strategies shows, that so far, a connection to a structural framework of reasons for PER constitutes a research gap, especially in relation to the inclusion of the use of digital media in these strategies.

### 2.4 Digital media use for PER

The utilization of digital media has experienced a notable surge in recent years, accelerated by the COVID-19 pandemic (10, 11). While stakeholders have advocated for this increased integration (12) a discourse concerning the pervasive implementation of digital media within daily educational practices has emerged (42). Research indicates that educators perceive novel pedagogical opportunities while simultaneously emphasizing the need for prudent application and consistent evaluation of digital media's impact (42). This discourse assumes heightened significance within PE, a discipline where sustained engagement in physical activity is paramount (32).

Recent research has broadly addressed digitalization in PE, particularly since the pandemic, which has accelerated the integration of digital media into education (43, 44). The concept of digital media is continually evolving (33). In this study, digital media encompasses all technological devices, software, and methodological settings used.

Teaching and learning often occur in digital settings, defined as “a generic term for online learning, digitally enhanced face-to-face learning, and blended learning, assuming that digital media are used as technology to enable or support the respective form of teaching” [(43), 4671]. This definition applies to PE when digital media are used. While research on digital media in PE has not focused on PER, some studies offer relevant insights. For example, Jastrow et al. (32) found that digital media use in PE is associated with increased student motivation. Mödinger et al. (45) demonstrated that digital media can support and enhance individual learning in PE, citing examples like movement learning and analysis using video feedback, potentially reducing PER. Similarly, Krick and Nowak (34) suggested that digital media can streamline organization and assessment, preventing general disruptions, and Raab (35) argued for their effectiveness in teaching exercise- and sports-related knowledge. These findings suggest that digital media may be relevant for teachers addressing PER. Conversely, Aagaard (36) highlighted teacher concerns about digital media as a source of distraction. The question of whether digital media offers more solutions or creates more problems remains open.

### 2.5 Research questions

Given that existing research on PER strategies lacks an overview and does not consider digital media, this study investigates such strategies in general and whether the use of digital media enables new strategies or modifies existing ones. Specifically, this study addresses the following research questions:

RQ1: What strategies do teachers use to deal with PER?
RQ2: How can digital media be used to deal with PER?

## 3 Method

This study adopts an exploratory approach (37) to address the research questions, aiming to understand the realities of daily PE. Gathering information from participants within a school setting is crucial for this purpose. Building upon existing teacher-focused research, this study employed interviews (37) with expert teachers regarding their experiences with PER. While other qualitative methods were considered, interviews were deemed most likely to yield rich insights. Eleven teachers were interviewed, some online and some in person. Interviews were recorded and transcribed following Kuckartz's (38) guidelines and analyzed using qualitative content analysis (39) with MAXQDA 2020 software.

### 3.1 Sample

All 11 participating teachers taught in the first and/or second levels of secondary schools in Germany. Other school types and age groups were excluded to ensure comparability of PE settings. Table 1 presents the interviewees’ sociodemographic data. Interviews, conducted by one author, ranged from 18 to 44 min in length.

**Table 1:** Sociodemographic data of interviewees.

Alias	Gender	Year of birth	Years of experience	Subjects taught (Excluding PE)
B01	Male	1987	9	Mathematics
B02	Male	1995	2	Physics
B03	Female	1970	24	Mathematics; Chemistry; Computer Science
B04	Female	1985	2	German; Mathematics; French
B05	Male	1992	2	Geography; German; Business, career- and study orientation
B06	Male	1985	11	Mathematics
B07	Male	1968	22	Science, Technology, Engineering and Mathematics; Geography
B08	Female	1994	3	Mathematics
B09	Male	1971	22	Biology
B10	Male	1985	10	Biology; Science, Technology, Engineering and Mathematics
B11	Male	1986	10	Ethics; Philosophy; Social Studies; Economics

All participants gave their written consent before the study, they were introduced to the research design and intended use of their answers before the interviews started, their confidentiality was ensured, and they were informed that they had the option to withdraw at any given time without the occurrence of any disadvantages.

### 3.2 Interview guide

Following best practices for expert interviews (40, 41), a semi-structured interview guide was developed. This approach allowed for structured conversation while providing flexibility for in-depth probing and ensuring comparability across responses. The interview guide, in reference to Misoch (41), began with informing interviewees about data protection rights and providing background information about the study. The core of the interview focused on the research questions. While the main questions remained consistent across interviews, additional, context-specific questions were incorporated as needed. This standardization ensured comparability, while individualized questions facilitated detailed responses. The interview concluded with collecting sociodemographic data and addressing any remaining questions.

### 3.3 Analysis

Qualitative content analysis aims to systematically, using established rules and theory, analyze conversations to identify emergent themes (39) and integrate findings with existing PER research. The well-defined theoretical framework of this study justified the use of qualitative content analysis according to Mayring (39), rather than Kuckartz and Rädiker (38). The data were organized according to empirically and theoretically relevant aspects to enable structured presentation of results (39). Structured categories were developed and used to code the interviews. The process of category development, coding, and analysis was iterative, continuing until no new categories or data emerged. This iterative approach allowed for both deductive and inductive category development (39). Tables 2, 3 display the categories and sub-categories. First-order categories were derived from the research questions, while sub-categories were drawn from existing literature and developed inductively during the analysis process (italicized in the tables).

**Table 2:** Categories for strategies teachers Use to deal with PER.

Category	Sub-category	Sub-sub-category
Strategies teachers use to deal with PER	Attitude toward PER	*Capacity*
		*Perception*
	Strategies in institutional dimension	School guidelines
		Collegial exchange
		*Work with parents*
	Strategies in classroom dimension	Rule setting
		Variety in scenarios/motivation
		Differentiation
		*Performance and evaluation*
		*Division*
		*Inclusion of passive students*
	Strategies in relationship dimension	Classroom climate
		Teacher behavior
		Interpersonal relationships

**Table 3:** Categories of digital media and their use for the prevention and intervention of PER.

Category	Sub-category	Sub-sub-category
Digital media used to prevent and/or intervene in PER	Movement learning	Movement analysis
		Practice template
		Gamification
	Performance assessment	–
	Organization	*Documentation*
		*Team division*
	Teaching sports-related knowledge	*Content participation*
	Involvement of passive students	*Exercise development*
		*Assistance*

## 4 Findings

### 4.1 Teachers’ strategies

Teacher strategies were categorized according to their respective dimensions: institutional, classroom, and relational (9).

#### 4.1.1 Strategies in the institutional dimension

In some cases, teacher actions were **dictated by school policy**. For example, late arrivals were documented (B05), and absences from PE required parental notes (B07). One teacher reported assigning copying text tasks to inactive students (B04). These strategies are based on pre-established institutional rules.

Notably, none of the teachers reported **collaborating with colleagues**, a strategy mentioned in the literature. One teacher expressed a need for such collaboration, stating, “because as a teacher, you are usually on your own” (B03), indicating a desire for institutional support.

One teacher **engaged with parents** to prevent PER, particularly regarding swimming lessons. He explained, “so, in swimming, we really try to prevent this [PER] through parents’ information evenings where we ask parents to send their children to us regularly for swimming training” (B07). This strategy addresses the issue at a higher, out-of-classroom level.

#### 4.1.2 Strategies in the classroom dimension

Teachers recognized the importance of varied learning opportunities, differentiation, and incorporating student interests to prevent PER. They reported creating diverse activities to challenge and support every student (B06), noting the need to address both high and low achievers to avoid boredom (B09). Some students might withdraw due to fear of certain exercises (B07), which teachers addressed through methodical instruction (B06, B08). One teacher explained,

if a child is incredibly afraid of doing a somersault, […] then maybe the child won’t do a somersault, but maybe they can do something else great in that area (B07).

**Referencing student interests** increased engagement and motivation (B04), although teachers acknowledged limitations imposed by the curriculum. One teacher explained the need to justify curricular choices: “why this [topic] or that [method] is following [now] or why this [topic] or that [method] is not following [immediately]” (B01), highlighting the interplay between institutional and classroom dimensions.

Teachers **emphasized the multifaceted nature of PE**, beyond just performance: “that it is not just about performance, as PE simply has more aspects” (B06), such as “healthy lifestyle” (B06), “fun and games” (B06), and overcoming fear (B02). One teacher advocated for reducing performance pressure:

[We should be] focused more on small successes as well, and the main thing is that the child gathers some experience of movement. I don’t think they have to get a grade for everything. That would take the pressure off many, many children (B07).

When grades were given, they included progress, motivation, cooperation, and social behavior (B05, B09).

When PER was attributed to group dynamics, teachers initially used r**andom group assignments, adjusting as needed** (B11). One approach to creating balanced teams was performance-based: “good ones playing against the good ones [and] the weaker ones playing against the weaker ones” (B03). Another teacher allowed students to self-select teams based on “sympathy” (B10), but still made adjustments if necessary.

To proactively prevent avoidance, one teacher aimed to **keep all students engaged**: “and if that doesn't work, then I try to give those who are not active at the moment some kind of assignment” (B10). Assignments included assisting with movement (B01, B02, B03, B05, B06, B10, B11), preparing equipment (B03, B11), writing reports (B06), conducting observations (B10), or refereeing/scoring (B11).

Regarding forgotten sportswear, some teachers (B05, B07, B08) **allowed participation when possible**:

Basically, if someone has just forgotten their sportswear, then they still take part in PE, even if it’s a gray area, of course. You’re not always allowed to do this for safety reasons; of course, it also depends on the sport. But it’s a practical way of preventing people from just saying, ‘I don’t feel like it’ and ‘I’ve forgotten my sportswear’ (B08).

#### 4.1.3 Strategies in the relationship dimension

One classroom strategy involved **establishing rules**. One teacher emphasized the importance of reinforcing agreements and “to set an example of a certain strictness or consistency,” to avoid student rebellion (B02). However, flexibility in applying rules was also recognized (B01), contrasting with institutionally defined rules and highlighting teacher-student collaboration in rule creation.

Structuring lessons around **basic and familiar concepts** was also recommended. For example, one teacher used familiar games to connect with and motivate students (B03), again demonstrating collaborative lesson development.

Teachers aimed to **create a positive classroom climate**, recognizing its influence on PER (B01, B06, B09, B11). This involved fostering a good atmosphere, a shared responsibility between teacher and students (B08), and preventing “derogatory comments or degrading behavior” (B11). Open communication between teachers and students, as well as among students (B06), and a “supportive culture” (B09) were prioritized. Building personal relationships was seen as essential for students to “open up and say what the problem is” (B04).

Teachers addressed barriers to interaction, such as shame, through **targeted interaction** (B06). One teacher described their approach to building rapport from the first lesson:

A concern for me [is] to be able to remember the names very quickly, to have the person in mind, and to be able to call them by name. I also always ask them what their favorite sport is, so that I can build a personal relationship (B02).

In cases of refusal, teachers **engaged in direct conversations** with students (B01, B02, B03, B04, B06, B08, B11) to “see what the reason is and take countermeasures accordingly” (B11). One teacher explained, “You have to be there immediately as a teacher and then defuse the situation and react to it” (B06), emphasizing the importance of immediate intervention and motivation. Teachers also stressed the **importance of their own motivation** (B04).

### 4.2 Digital media for dealing with PER

The digital tools mentioned by teachers are categorized by their application (see Table 3).

#### 4.2.1 Movement learning

Digital tools for movement learning included exercise templates provided via **digital station cards** or explanatory **videos** (B02, B05, B07) on **tablets** (B02, B05) or assigned for home viewing (B07). These templates provided variety, reduced downtime (B01), facilitated self-regulated learning (B04), and enabled differentiation (B05), all identified as potential triggers for refusal behavior. Careful attention to student-friendly language and appropriate level was emphasized (B01).

Video recordings with subsequent movement analysis as feedback were used to accelerate progress and increase motivation (B10). Video recordings also helped students correct their self-perception, reducing anxiety and facilitating learning (B02, B07). Standard camera apps on **smartphones**/**tablets** or **video delay programs** were used (B07).

#### 4.2.2 Performance assessment

One teacher suspected that performance pressure contributed to PER: “you don't want to present yourself in front of others in a sport where you are not so good” (B11). He suggested **video recordings** and **tracking apps** to alleviate this pressure:

If, for example, it’s about fear, like performing something in front of the group that is then graded, a video recording would be possible […] I did that once during [COVID-19], where the students used a running app to track what they practiced and things like that, and then, of course, that can be included in the assessment (B11)

This approach allowed performance assessment without direct social interaction.

#### 4.2.3 Organization

One teacher used a **tablet to record** forgotten sportswear (B01). **Online surveys** were used to gather student interests and inform lesson planning, reducing reasons for avoidance (B01, B02). The “Team Shake” **app** was used to quickly and easily create balanced teams, minimizing potential frustration (B01, B03, B11).

#### 4.2.4 Teaching sports-related knowledge

One teacher (B06) identified the **flipped classroom** as a preventive strategy against refusal behavior:

Many don’t have previous contact points to a certain kind of sport, so, via a video, for motivation, I can give them homework in preparation for the lesson. I make sure that I simply keep the students a bit interested and also inform them about some of the rules and regulations. Maybe also a bit of the history of sports (B06)

This approach addresses the theoretical aspects of sports. When refusal occurred, students could be meaningfully engaged with sports theory (B11). **Film or video content** on sports, rules, game tactics, or techniques could be shown or assigned as homework (B01, B03, B09). One teacher suggested using movement simulation **apps**, if available:

Such a task, I think, could also help people who refuse lessons to simulate certain movements on the iPad and present them to others. [This could] help them visualize and reflect on basketball [techniques] (B01).

#### 4.2.5 Involvement of passive students

Teachers frequently suggested auxiliary activities to involve refusing students (B02, B03, B05, B06, B08, B10, B11), including:

• Recording **videos** and **analyzing** movements with classmates (B02, B03, B05, B08, B10, B11);
• Refereeing, such as using **digital scoreboards** (B03);
• Taking **measurements** (e.g., speed or pulse) with **apps** and providing feedback (B06).

One teacher (B04) noted that students could create personalized training programs during class, drawing **inspiration from online resources** and “recording themselves or using apps in this regard” (B04).

## 5 Discussion

### 5.1 Enhancing existing systematizations

**Figure 2:**
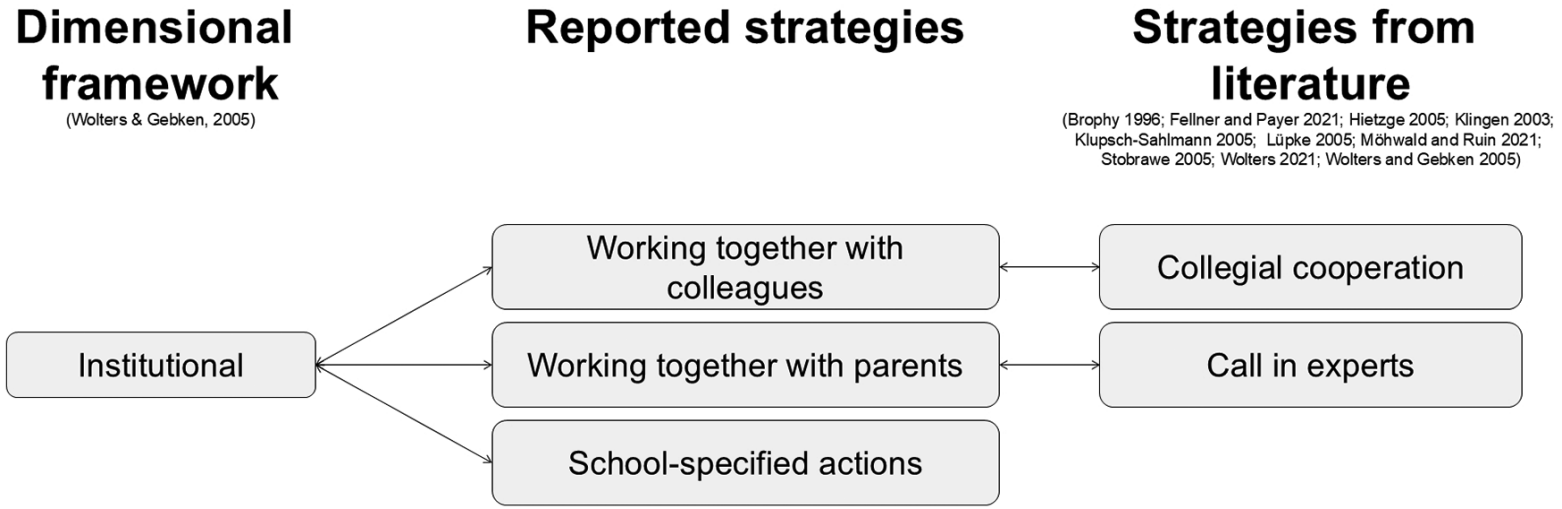
The empirical connection between Wolters and Gebken's (9) framework to strategies found in literature (9, 22–25, 27–31)—institutional dimension.

**Figure 3:**
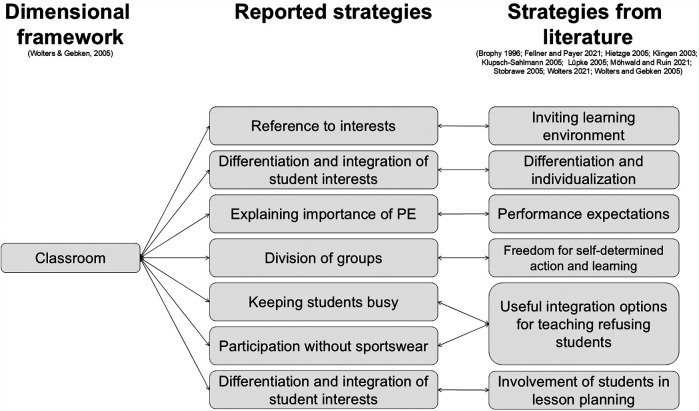
The empirical connection between Wolters and Gebken's (9) framework to strategies found in literature (9, 22–25, 27–31)—classroom dimension.

**Figure 4:**
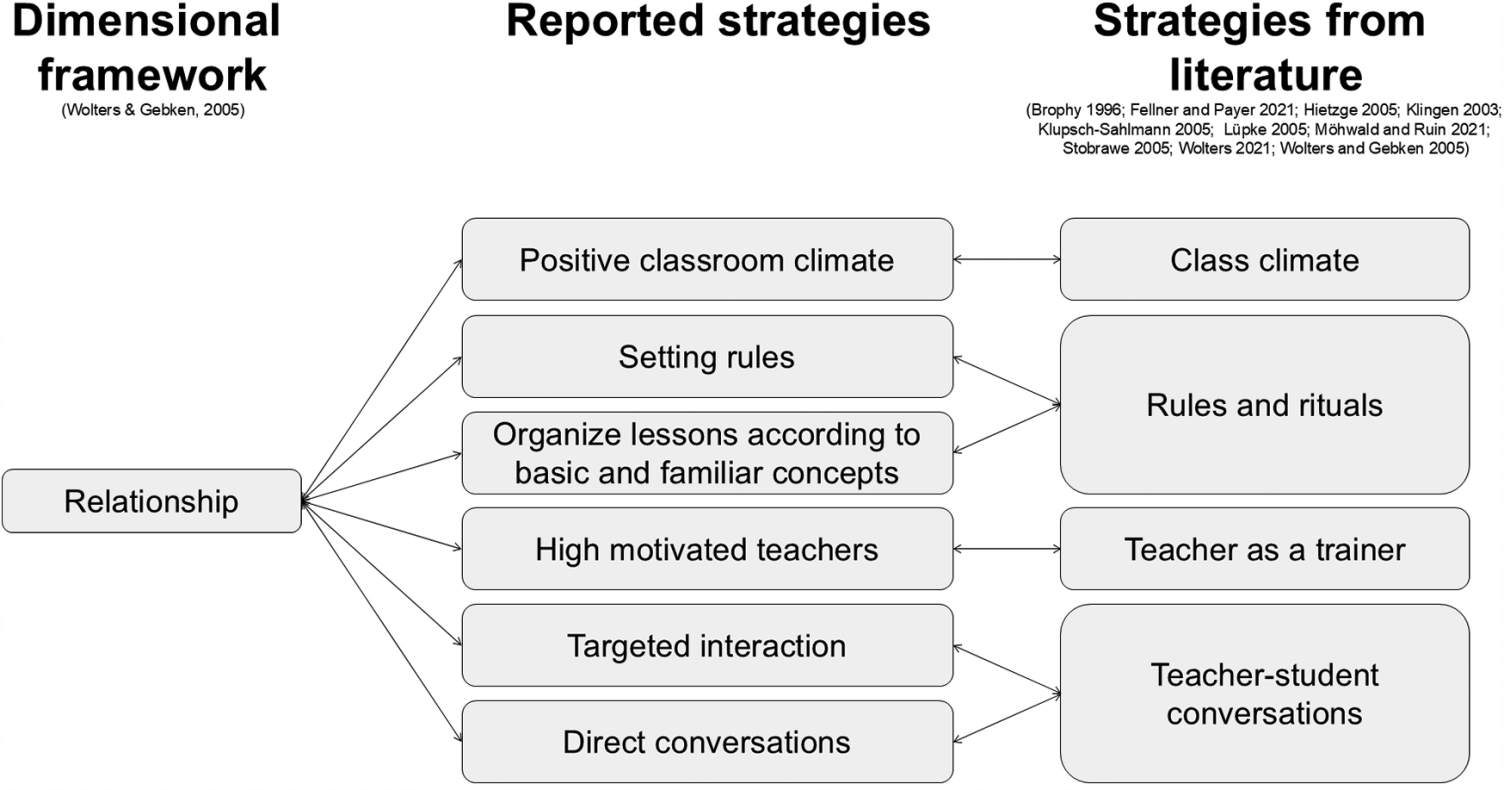
The empirical connection between Wolters and Gebken's (9) framework to strategies found in literature (9, 22–25, 27–31)—relationship dimension.

Without considering digital media, the existing PER framework by Wolters and Gebken (9), combined with strategies reported by various authors (9, 22–25, 27–31), provides a foundation for incorporating this study's findings. Teacher-reported strategies can be categorized within the institutional, classroom, or relationship dimensions. Furthermore, nearly all reported strategies align with strategies in the literature, thus connecting the collected strategies to the three dimensions. Figures 2–4 illustrate this concept and present a preliminary model.

The existing three-dimensional framework by Wolters and Gebken (9) effectively categorized the responses. Institutionally, collegial cooperation and expert consultation (9, 23) were reflected in **teachers working with colleagues and parents**. Conversely, teachers (B04, B05, B07) mentioned **school-specified actions**, which, while not connected to existing strategies, clearly belong to the institutional dimension, thus expanding the existing body of strategies. Seeing that school-specified actions are reported by the teachers but not supported by literature suggests that there are other, yet unknown, strategies for dealing with PER.

Within the classroom dimension, creating inviting learning environments (28) was linked to **teachers addressing student interests**. Differentiation and individualization (9, 22, 25) were evident in teachers’ **differentiated approaches** and **integration of student interests**. **Explaining the importance of PE**, as one teacher did by emphasizing movement experiences over grades (B07), relates to addressing performance expectations (22). Self-regulated **group division** (B10) reflects, in part, the freedom for self-determined action and learning (28). **Keeping students engaged**, even **without sportswear**, aligns with strategies for integrating refusing students (28–31). While **differentiation and interest integration** were previously categorized, they also relate to involving students in lesson planning (9, 23–25, 28). Interestingly, the classroom dimension includes more strategies than the previously discussed institutional dimension. It can be assumed that teachers have been able to acquire more strategies over time within their didactic and methodological possibilities for dealing with PER due to their experience. Further, it is possible that teachers expand their repertoire faster than new possibilities are communicated or provided by the school.

In the relationship dimension, the importance of a **positive classroom climate**, as highlighted by teachers, is consistent with Fellner and Payer (28). The teachers’ emphasis on **motivation** (B04) connects to teachers in function of a trainer (9, 29). Rules and rituals (9, 24, 28) were reflected in teachers **setting rules** and organizing lessons around **familiar concepts**. The teacher-student conversation (9, 22, 29) was evident in teachers’ **targeted interaction** and **direct conversations** with students. Teachers reported more strategies in the relationship dimension than in the institutional dimension, but fewer than in the classroom dimension. This difference likely derives from teachers having greater autonomy to shape their classroom environment and choose communication strategies compared to actions dictated by school policy. Wolters and Gebken’s (9) framework positions the three dimensions as equal. However, regarding influence, these dimensions likely differ for teachers. Teachers presumably have the most direct influence on the classroom dimension, as they are responsible for creating and managing the didactic setting. Their influence on student interaction is shared with the students themselves. Furthermore, teacher influence appears to diminish when strategies are predetermined by school authorities. This study did not explicitly address this concept, suggesting a potential avenue for future research.

Interestingly, a variety of methods (28) and appropriate responses to disruptions (9, 27, 29) were not explicitly mentioned. This may be due to their implicit nature. Variety of methods is inherent in differentiated approaches, and responding to disruptions is central to the study's focus. Therefore, the relevance of these strategies is not in question.

This overview addresses RQ1, demonstrating the diverse strategies teachers use, aligning with existing systematizations in the literature. This study represents the first attempt in international research to connect Wolters and Gebken's (9) framework with a collection of strategies from the literature and support this connection with empirical findings. The resulting preliminary model can serve as a foundation for future investigations, though it is not exhaustive. Critically, it enables linking the complex set of strategies for dealing with PER to other areas, such as digital media use.

### 5.2 Dealing with PER through digital media

As discussed, teachers use diverse strategies to manage PER. Digital media can support or enable some of these strategies. Examining this connection, it can be seen that arguments were primarily made within the classroom dimension. No reported digital media directly connected to institutional or relational strategies. Therefore, only the classroom dimension and its strategies will be focus of further discussion.

Teachers use the term “digital media” broadly and adapt its application to specific situations, consistent with previous research showing that teachers expand and optimize existing strategies and views through digitalization (42). They appear less strict in their selection and application of digital media as long as it achieves the desired outcome: reduced PER. As the term digital media was not defined prior to interviews, teachers mentioned diverse types: hardware (**tablets, smartphones, digital boards**), software (**digital station cards, videos, video delay programs, apps, online surveys, internet use**), and methods like **flipped classrooms**. Some of these align with existing research: Mödinger et al. (45) research video use on tablets; Krick and Nowak (34) focus on smartphone and tablet apps; and Raab (35) relates to methodological choices like flipped classrooms or internet research. Having a closer look at the different categories of digital media mentioned by the teachers, it can be seen that the responses are consistent with current literature. Hardware like tablets, smartphones, and digital boards can support creating inviting learning environments and provide opportunities for self-directed learning. They can also facilitate integrating typically passive students, encouraging active participation and potentially reducing PER (9). Software like digital station cards allows for self-determined action and learning (28) while creating an inviting learning environment. Combined with apps for tracking, measuring, or organizing (34), they support strategies like addressing performance expectations or integrating students. Videos, as mentioned by Mödinger et al. (45), were seen as motivating. Recording and analyzing videos connects to multiple strategies: creating an inviting environment, differentiation, individualization, and integration. It also provides transparent performance feedback. Online surveys (B01, B02) can actively involve students in lesson planning, while internet research allows for planning involvement, participation even without sportswear, and self-directed learning. Teachers see diverse possibilities in using software to address PER. Flipped classrooms (B06) occupy a unique position. As a methodological teaching approach, it raises the question of whether it constitutes a strategy itself. As a digital medium in this study, it allows students to prepare for lessons and performances beforehand, self-direct their learning, and participate in PE without directly performing. Therefore, flipped classrooms can also be used to address PER. Table 4 summarizes the extracted connections between digital media and classroom strategies.

**Table 4:** Digital media used in various strategies for dealing with PER.

Strategy used	Digital media used							
	Tablet/Smartphone	Digital boards	Digital station cards	Video (Delay program)	Apps	Online survey	Internet research	Flipped classroom
Inviting learning environment (28)	X		X	X				
Differentiation and individualization (9, 22, 25)				X				
Performance expectations (22)				X	X			X
Freedom for self-determined action and learning (28)	X		X	X	X		X	X
Useful integration options for teaching refusing students (28–31)		X		X	X		X	X
Involvement of students in lesson planning (9, 23–25, 28)						X	X	

**Figure 5:**
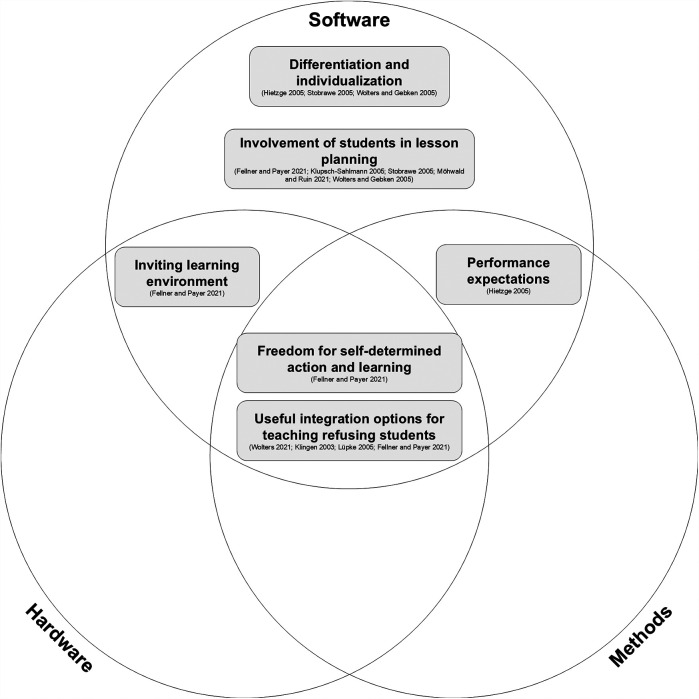
Classroom dimension strategies for dealing with PER systemized by digital media category.

Restructuring Table 4 to focus on method, hardware, and software reveals that all strategies in the classroom dimension were associated with software use. Some strategies also involved hardware and/or the flipped classroom method. Thus, the interviewed teachers demonstrated the highest strategic diversity using software to address PER. While combinations with other categories are evident (Figure 5), it is notable that no classroom strategy appears to be implemented exclusively through hardware or flipped classrooms. Teachers can consistently address how digital media are used to deal with PER through software and expand from there. While the preliminary model for strategies (Figures 2–4) showed teachers perceiving their highest strategic influence in the classroom, this specialized analysis of digital media influence suggests a preference for software solutions. Similar to the distribution of general strategies, this may be related to the ease of access. Depending on existing knowledge and comfort with digitalization, accessing and experimenting with new software may be simpler for teachers than, for instance, acquiring new hardware. As exploratory research, this study not only lays the groundwork for model generation regarding general PER strategies, but also sets the stage for future research examining the concept and selection of digital media in greater detail.

Within a broader educational context, the applicability of this concept extends beyond PE. As previously demonstrated, teachers’ integration of digital media is primarily guided by pedagogical intent rather than subject-specific constraints. Research across diverse disciplines underscores the perceived criticality of effective digital media utilization (42). Examining the deployment of digital media to address PER corroborates this consensus among PE instructors. Despite the unique characteristics often attributed to PE, this study reveals a consistent influence of digitalization across subjects, necessitating a comprehensive discussion of the general impact of digital media within education.

Consistent with the observed comparability to general education and digital media, interviewed teachers acknowledged potential drawbacks. Yet, the inherent tension between physical activity and screen time was not perceived as problematic, as teachers emphasized the intentional use of digital media to augment movement opportunities. While some teachers noted a potential increase in refusal behavior when digital media options were available (e.g., video review filming), this was not attributed to digitalization itself. Rather, refusal was linked to potential external distractions overshadowing lesson engagement. Notably, infrastructural limitations, such as connectivity or device issues, were not cited. The study's design, focusing specifically on PER, likely influenced this emphasis.

### 5.3 Limitations and future directions

The answers of 11 participants allow for generating insights into PER, particularly in conjunction with digital media. Due to the low number of participants, this exploratory approach cannot provide definitive answers, but interviewing teachers confirmed and expanded current research, linking teacher responses to digital settings. However, the often-criticized focus on teachers in classroom management and disruption research (42, 43) calls for incorporating student and stakeholder perspectives in future research. Especially the perspective of students will be beneficial to investigate in this regard since PER addresses student behavior and should therefore include the perspective of the students in question. Furthermore, it is important to consider follow-up quantitative evaluation studies and modifications of the derived model approach. The discussion was able to develop model approximations that require further refinement. Not only is a larger data basis required for this, but a clearer definition of the term digital media is also necessary for a more detailed consideration of the topic and the development of larger models.

As seen in the framework, international research on the topic of PER is missing. This study, conducted in Germany with only German teachers provides an opportunity to broaden the field into international research, urging for international enhancement. It is important to acknowledge differences in educational systems worldwide. Combining decades-old research on classroom management and disruptions with new digital possibilities means that differences in educational systems, social structures, stakeholder priorities, and other factors significantly impact this topic. Therefore, this study leaves questions open for future international research. Even a closer look at the German system reveals gaps, such as different school types, student age groups, or participant social backgrounds. This already becomes clear within this study by the fact that the question of why teachers used certain digital media for certain strategies cannot be answered. Future research can start here.

As the discussion illustrates, existing research can be synthesized. However, it also reveals that Wolters and Gebken's (9) framework can be challenged regarding a potential hierarchy of the dimensions based on teacher influence. While this study's focus and data cannot yield clear answers to this, it offers a starting point for future research. Subsequent research can build upon the model approximations presented in the discussion to develop practical tools for teachers. A more precise definition of digital media, as previously mentioned, is one possible direction for concretizing these tools. Incorporating the learners’ perspective, in addition to the teachers’, could also be valuable in this context.

Finally, this study could not determine whether digital media are more beneficial or a greater source of disruption. As existing research suggests, choosing the right approach and focusing on creating a beneficial learning environment for all participants is crucial. Using digital media simply because it is available should be carefully considered (42).

## 6 Conclusion

Physical education refusal (PER) is a subject-specific type of classroom disruption (9, 24). Effective classroom management, including structured approaches to disruptions, is crucial for enhancing learning outcomes (2) and supporting teacher well-being (6, 7). Teacher responses to PER typically fall within institutional, classroom, and relationship dimensions (9). Various authors (9, 22–25, 27–31) have reported potential strategies for addressing PER. This study provides the first empirical basis in international research for linking these strategies to the dimensional framework. The resulting preliminary model can inform future research. Furthermore, existing research has not fully explored the increasing significance of digitalization. This study offers novel insights into teachers’ understanding of digital media and how they implement PER strategies using these tools. In general, teachers tend to favor classroom-based strategies within their methodological-didactic approach, often implemented through software solutions. These findings highlight several avenues for future research, including cross-cultural comparisons of educational systems, further model refinement, and the development of practical guidance for teachers.

## Data Availability

The raw data supporting the conclusions of this article will be made available by the authors, without undue reservation.
